# Effects of Starve and Shelter Availability on the Group Behavior of Two Freshwater Fish Species (*Chindongo demasoni* and *Spinibarbus sinensis*)

**DOI:** 10.3390/ani14162429

**Published:** 2024-08-22

**Authors:** Wuxin Li, Jiaqian Li, Shijian Fu

**Affiliations:** Laboratory of Evolutionary Physiology and Behavior, Chongqing Key Laboratory of Conservation and Utilization of Freshwater Fishes, Animal Biology Key Laboratory of Chongqing Education Commission of China, Chongqing Normal University, Chongqing 401331, China; 2023010513007@stu.cqnu.edu.cn (W.L.); 2019110513053@stu.cqnu.edu.cn (J.L.)

**Keywords:** fasting, simulated predation attack, shelter utilization, interspecific differences, dynamics

## Abstract

**Simple Summary:**

Anti-predator behavior is an important means for fish to avoid predator attacks. This study investigated the anti-predation strategies of qingbo (*Spinibarbus sinensis*) and demasone cichlid (*Chindongo demasoni*) in a six-arm maze with different arm types, examining both fasted and non-fasted states. We found that in the non-fasted condition, both species chose to aggregate into shoals and then enter the shelter to hide when a shelter was present in the environment, whereas, in the absence of shelter, qingbo still chose to enter the arm and demasone cichlid chose to congregate in the central area. This difference in the selection of anti-predator strategies was even more pronounced in the fasted state. This may foreshadow interspecific differences in the effect of fasting on anti-predation strategies and indicate that the availability of shelter has a non-negligible effect on this.

**Abstract:**

In complex environments, fish often suffer from reduced physiological functioning due to starvation, which may have a significant effect on their behavioral adaptive strategies to predator attacks. We selected qingbo (*Spinibarbus sinensis*, which prefers flowing water habitats) and demasone cichlid (*Chindongo demasoni*, which prefers still water habitats), to investigate the differences in group distribution and dynamics between the two species when faced with a simulated predation attack under different trophic states (fasted for 2 weeks or fed). We chose to conduct our experiments in a six-arm maze that included a central area and six arms of equal length and width and to obtain evidence of how the fish used the various areas of the maze to respond to simulated predation attacks. We found that the two fish species differed in their responses to simulated predation attacks under different trophic states. The group structure of the two species was relatively stable, and the effect of fasting on the qingbo group was not significant, whereas the demasone cichlid group was more susceptible to the effects of fasting, shelter and a simulated predation attack. In an environment with shelter, both species had the same anti-predator strategy and tended to enter the shelter arm to hide after encountering a simulated predation attack. However, differences in the anti-predator strategies of the two species emerged in the no-shelter environment, with the qingbo tending to enter the arm to hide, whereas the demasone cichlid group chose to enter the central area to congregate, and this phenomenon was more pronounced in the fasted group. In conclusion, our research shows that even group-stable fish may shift their anti-predation strategies (i.e., entering a shelter to hide shifts to aggregating in situ into a shoal) when starved and that the worse the swimming ability of the fish, the more affected they are by starvation.

## 1. Introduction

Predation is a key factor in the natural selection of prey species [1]. In response to the predation risks that they encounter in complex and unstable environments, fish frequently adopt adaptive strategies, including morphological, physiological and behavioral phenotypes [2]. Fish behavior in the face of predation pressure is often characterized by reduced activity and the increased use of shelter [3,4,5]. The common roach (*Rutilus rutilus*) has been shown to increase the use of shelter to escape predators [6]. Furthermore, when fish are faced with a predator attack, their primary behavioral strategy is grouping [7]. Most fish live in groups in their natural environments, and this lifestyle persists throughout all or most of their lives [7]. However, the structure of shoals is not static and can change in response to environmental changes [8,9]. It has been proposed that, among different species, differences in predation pressure, food availability and ecological habits lead to differences in group ecological benefits, group living costs and spatial distribution patterns [10,11]. Meanwhile, fasted fish adjust their predator inspection and foraging behavior in response to increased predation risks, which may differ from that of fed conspecifics [12]. We believe that the choice of this behavioral strategy may be inconsistent across different habitats (i.e., presence and absence of shelters) and between different fish species from different habitats or in different physiological states.

In nature, factors such as environmental change and seasonal variation often result in significant spatial and temporal heterogeneity in the food available to animals, and they may suffer from starvation to different extents [13,14], which may have a strong effect on their ability to respond to predation risks [15]. Starvation has been shown to affect energy metabolism [16,17], physiology and biochemistry [18], phenotypes [19] and personality [20,21], among other factors. This suggests that fish adapt their physiological, biochemical and behavioral strategies in response to fluctuations in food abundance (e.g., reducing physical activity, decreasing levels of body energy metabolism and increasing foraging ranges) [22]. When the energy reserves and metabolic status are altered, the locomotor ability is directly affected [23]. Animal locomotor strategies (e.g., intermittent motion and freezing) have been shown to favor prey survival [24], and the selection of locomotor strategies under starvation may be crucial. We believe that a starvation-induced reduction in fish body functions may reduce the probability of escaping from predators, which may be fatal for fish, and starved fish may change their behavioral strategies to escape from predators.

We selected two species (qingbo, *Spinibarbus sinensis*, and demasone cichlid, *Chindongo demasoni*) to investigate the group distribution and dynamics before and after attacks by a simulated predator performed under different trophic conditions. Qingbo is a common benthic omnivorous fish of the family Carpidae in the Yangtze River system in China, usually living in the flowing areas of the mainstem and tributary systems of the Yangtze River [25]. Demasone cichlid is a common tropical ornamental fish native to Lake Malawi, usually living at the bottoms of lakes and rivers [26,27]. The habitats of qingbo and demasone cichlid are complex, and there are differences in their swimming abilities. Starvation may not impose the same level of restriction for the two species. We believe that starvation affects species with different locomotor abilities to different extents.

## 2. Materials and Methods

### 2.1. Fish and Animal Ethics

In total, 400 demasone cichlid fish were purchased from the Shapingba Fish Market, Chongqing, China, and 400 qingbo fish were purchased from the Hechuan Aquaculture Base, Chongqing, China. After transfer, the two species were acclimated in two water-circulating tanks (approximately 250 L) for two months. Each species was allocated a water-circulating tank and the two species were not mixed for acclimation. Ten green plastic aquatic plants (height: 30 cm) were randomly placed in each tank to increase the environmental richness. During the acclimation period, the fish were fed once a day with frozen bloodworms at 10:00 am. To reduce the influence of oxygenation and the circulating water flow on the feeding of the fish, the air pump was turned off for 10 min before each feeding. After one hour of feeding, residual food and feces were removed using a siphon to maintain the water quality of the tank, which decreased the water volume of the tank. Hence, approximately 15% of the tank water was supplemented with dechlorinated fresh tap water every day. The dissolved oxygen level was maintained above 7.0 mg/L via an air pump with a 14 L:10 D photoperiod. The water temperature of each tank was maintained at 25.0 ± 0.1 °C using a temperature controller.

In this study, all animal handling and experiments were conducted in strict accordance with both the ethical requirements and the recommendations for animal care of the Key Laboratory of Animal Biology of Chongqing, China (Permit No. Fu2020112302) and the requirements for environmental and housing facilities for laboratory animals in China (GB/T14925-2001). All experiments also complied with the local animal welfare laws (i.e., the measures of the Chongqing municipality for the administration of experimental animals) of Chongqing City, China.

### 2.2. Experimental Overview

After acclimation, 256 juvenile qingbo (body weight: 2.00 ± 0.06 g; body length: 4.88 ± 0.04 cm) and 256 juvenile demasone cichlid (body weight: 1.66 ± 0.54 g; body length: 4.21 ± 0.54 cm) fish were randomly selected from the fasted group (fasted for 2 weeks) and the fed group (normal fed). A total of 64 shoals (group members: *N* = 8) were formed, and 32 shoals were used in Experiments 1 and 2, respectively. Because the sex of the fish could not be distinguished from their external morphology, male and female fish were randomly assigned to each treatment group. Our study included two experiments. Experiment 1 was designed to investigate the group changes in the two species with different trophic statuses in the face of a simulated predation attack under test conditions with (i.e., one shelter arm and five normal arms) and without (i.e., six normal arms) shelter (a green plastic plant, approximately 20 cm in diameter). Experiment 2 was designed to investigate the group changes in the two species with different trophic statuses before and after attacks by a simulated predator performed under test conditions with shelter (i.e., one shelter arm) and food (i.e., one food arm, 10 bloodworms).

### 2.3. Experimental Devices and Measurement Methods

**Figure 1:**
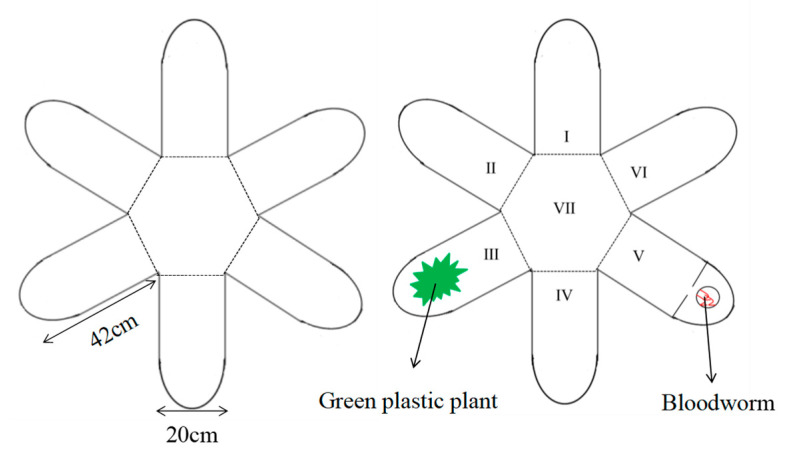
Schematic drawing of the 6-arm radial arm maze. I, II, IV, and VI: normal arms; III: shelter arm; V: food arm; VII: center area. For each test, the arms were chosen randomly to serve as the shelter arm or food arm.

In this study, we used a 6-arm radial maze (consisting of a central area and 6 arms of equal length and width; see Figure 1 for details) to measure fish group behavior. The center area was a square hexagon (side length of 20 cm), and each arm (42 cm length × 20 cm width × 20 cm height, water depth of 15 cm) was constructed from a 1.5-cm-thick nontoxic white PVC sheet. To eliminate the reflective effect of the inner walls of the apparatus on the fish, the bottom and inner walls of the maze were covered with white nontoxic opaque paper. The water temperature (25 ± 0.1 °C) and dissolved oxygen concentration (7.0 mg/L) in the maze were consistent with those in the acclimated environment. A high-definition wide-angle camera (Logitech C920, Logitech, China, 30 frames/s) connected to a computer was installed directly above the experimental area to measure the behavior of the fish in all areas of the 6-arm maze.

The experimental steps were as follows. The test shoals consisted of 8 fish that were transferred to an opaque tank (approximately 15 cm in diameter) for acclimatization for 10 min. The species were tested separately. The fish were not exposed to air during the transfer process to prevent them from being stressed by air exposure. After acclimatization, the acclimatization device was slowly removed, and the fish were continuously filmed for 20 min. An egret (*Egretta garzetta*) model (body length 28 cm) attached to the end of a metal rod was manipulated to hover and dive above the maze during the 17th minute of filming to simulate a predator attack on the fish, which lasted for 10 s. The filmed videos were converted into an appropriate format and analyzed using MATLAB (vR2020b) to obtain the position of each fish, which was used to assess the number of individuals contained in each area of the maze (central area and each arm). The filmed videos were divided into two phases: the first 10 min reflected the group dynamics of the different fish groups under undisturbed conditions, while the data before and after 1 min of simulated predation attack were used to compare the effects of the simulated predation attack on the group dynamics of the different fish groups [28]. We defined the state of the fish before the simulated predation attack as the natural state and the state of the fish after the simulated predation attack as the stress state.

### 2.4. Data Collection and Calculations

In this experiment, we extracted 2 types of parameters (density and group dynamic parameters). The density was the average number of individual fish per frame in each region (shelter arm, normal arm and central area). The dynamic parameters of the fish group included the *I_c_* (cohesion index), the grouping frequency, the duration of the group and the percentage of time in the group. Fish clusters were determined as follows: if the number of individuals in an arm was ≥*N*/2 + 1 (*N* = 8), the current group of fish was considered to be in a group [29]. The grouping frequency (times/min^−1^) was the number of times that a fish reached a group per unit of time. The duration of the group (s) was the total time taken to reach the group state. The percentage of time in the group (%) was the percentage of grouping time out of the total measurement time (10 min). *I_c_* indicated the ability of animals to form cohesive groups in the radial maze. *I_c_* varied between 0 and 1, increasing as the number of occupied zones decreased and the groups became larger. The calculation formula for the parameters is shown below [29].

$$I_{c}=\frac{D_{c}-D_{min}}{N-D_{min}}$$

where *D_c_* is the Euclidean distance between the number of individuals (*f_i_*) in each area of the maze and *D*_min_ is the minimum *D_c_* value, i.e., the value of the fish group in its most dispersed state. *N* is the total number of fish. The calculation formula for *D_c_* is shown below.

$$D_{c}=\sqrt{\sum_{i=1}^{N}{\left(f_{i}\right)}^{2}}$$

where *f_i_* is the number of individuals per area in the maze. N is the total number of fish.

### 2.5. Statistical Analysis

Microsoft Excel (v2010) was used for the basic calculation of the data, and SPSS (v19.0, SPSS, Inc., and IBM, Chicago, IL, USA) was used for all statistical analyses. All data are expressed as the mean ± SE, and the significance level for all tests was set as *p* < 0.05. All data were first tested for normality and homogeneity of variance using the Kolmogorov–Smirnov and Levene tests.

We then used a mixed linear model ANOVA to test how predation, fasting and shelter influenced the shoals (e.g., *I_c_*, grouping frequency, duration of group and percentage of time in group). This mixed model included predation, fasting and shelter as fixed categorical effects; the number of fish as a random categorical effect; and the *I_c_*, grouping frequency, duration of group and percentage of time in a group as dependent variables. We then used the mixed linear model ANOVA to test how predation, fasting and shelter influenced the distribution of the shoal (e.g., the density in the arm and the density in the center area). This mixed model included predation, fasting and shelter as fixed categorical effects; the number of fish as a random categorical effect; and the density in the arm and the density in the center area as dependent variables. We then used the mixed linear model ANOVA to test how predation, fasting and the arm type influenced the density and shoal (e.g., arm density and percentage of time in group). This mixed model included predation, fasting and the arm type as fixed categorical effects; the number of fish as a random categorical effect; and the arm density and percentage of time in a group as dependent variables. If this model reported differences among or within the categories, Duncan’s multiple comparisons were used to test the significance of the differences when they were significant. Differences in the density distribution of fish in the different arms were tested using Fisher’s precision probability test.

## 3. Results

### 3.1. Effects of Simulated Predation Attack, Fasting and Shelter on Dynamics

**Figure 2:**
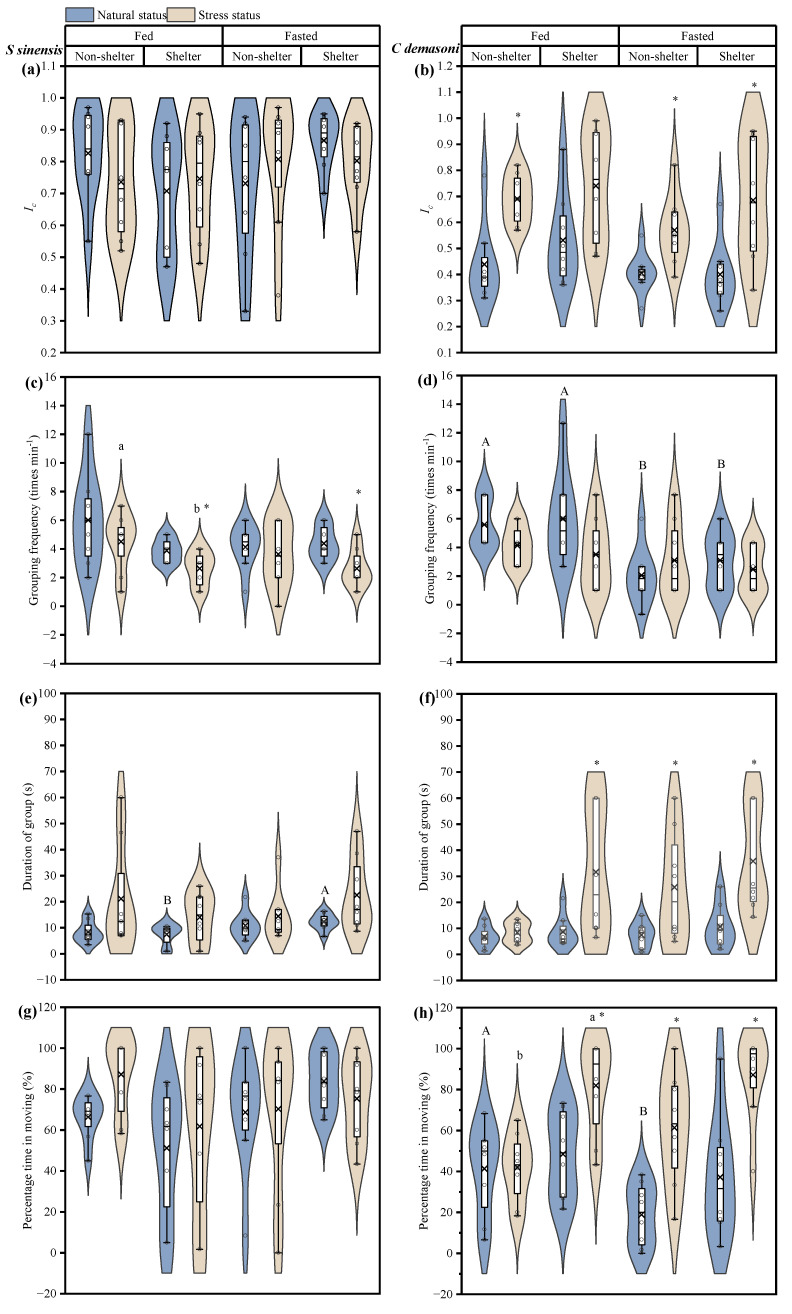
Effects of simulated attack, fasting and shelter on the dynamics of *Spinibarbus sinensis* (**a**,**c**,**e**,**g**) and *Chindongo demasoni* (**b**,**d**,**f**,**h**). This figure is a box diagram plus a violin diagram. The black circle (o) indicates the data. The fork (×) indicates the mean of the group. The black horizontal line (―) indicates the median of the group. The widening part of the violin diagram indicates that more data are present in this area. * indicates significant differences between natural and stress states (*p* < 0.05); a, b indicate significant differences between measurements in the maze with and without shelter (*p* < 0.05); A, B indicate significant differences between the fed and fasted groups (*p* < 0.05).

In qingbo, the *I_c_* of the fasted and fed groups did not change significantly before and after the simulated predation attack, either in the shelter or no-shelter environment (Table 1, Figure 2a). This suggests that group cohesion in qingbo is not affected by fasting. However, in demasone cichlid, the *I_c_* increased significantly (*p* < 0.001) after the simulated predation attack (Figure 2b). In addition, the *I_c_* was lower in the demasone cichlid fasted group than in the fed group (*p* = 0.038).

In qingbo, the simulated predator attack resulted in a significant decrease in the grouping frequency (*p* = 0.003) but an increase in the duration of the group (*p* = 0.003) (Table 1, Figure 2c,e). However, there were no significant differences in the percentage of time spent moving for each treatment (Table 1, Figure 2g). Moreover, the frequency of movement of the qingbo shoal was not affected by fasting. In demasone cichlid, only the fed group in the no-shelter environment was unaffected by the simulated predation attack, but, in the presence of shelter, both the duration of the group and the percentage of time in the group significantly increased after exposure to the simulated predation attack (Figure 2d,f,h). However, the duration of the group and the percentage of time in the group after exposure to the simulated predation attack increased significantly (*p* < 0.05) in the fasted group, both with and without shelter in the environment. In addition, before the simulated predation attack, the grouping frequency of the fasted group was significantly lower than that of the fed group under both the shelter and no-shelter conditions, and the percentage of time in the fasted group was significantly lower than that in the fed group under the no-shelter condition (*p* < 0.05).

### 3.2. Effects of Simulated Predation Attack, Fasting and Shelter on Distribution

**Figure 3:**
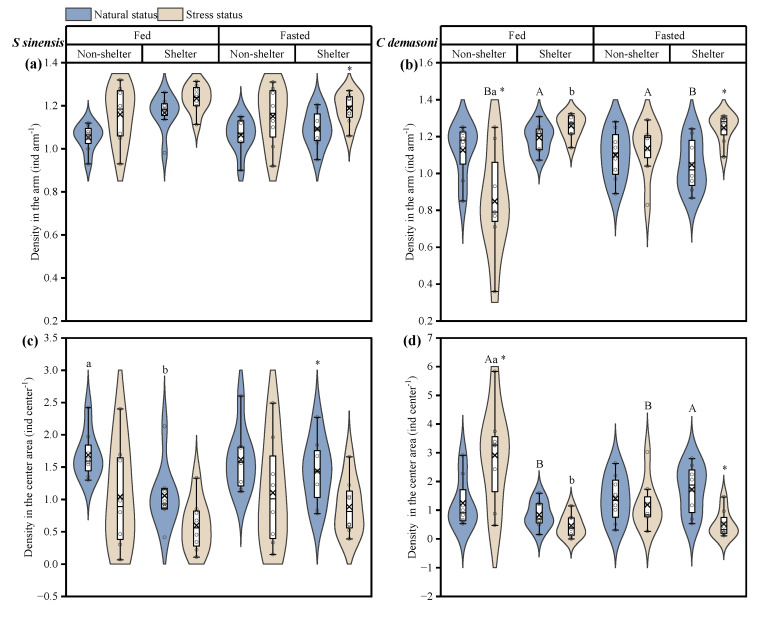
Effects of simulated attack and fasting on the distribution of *Spinibarbus sinensis* (**a**,**c**) and *Chindongo demasoni* (**b**,**d**). This figure is a box diagram plus a violin diagram. The black circle (o) indicates the data. The fork (×) indicates the mean of the group. The black horizontal line (―) indicates the median of the group. The widening part of the violin diagram indicates that more data are present in this area. * indicates significant differences before and after the simulated predator attack (*p* < 0.05); a, b indicate significant differences with and without shelter (*p* < 0.05); A, B indicate significant differences between the fed and fasted groups (*p* < 0.05).

In qingbo, the density in the arm increased and the density in the center area decreased after the simulated predation attack (*p* = 0.001) (Table 2, Figure 3a,b), and there was a significant difference in the fasted group before and after the simulated predation attack in the shelter environment. This difference was also present in the fed group before the simulated predation attack (*p* = 0.007) (Table 2, Figure 3a,b). In contrast to qingbo, after the simulated predation attack, the density in the arm of demasone cichlid was significantly increased, but the center density increased in the fed group in the no-shelter environment (Figure 3c,d). Shelter availability had a significant effect on the group distribution (*p* = 0.003), while it interacted with the feeding treatment, showing a significant difference only in the fed group after the simulated predation attack. In addition, before the simulated predation attack, the density was greater in the arms and lower in the central area in the shelter environment than in the fed group. After the simulated predator attack, the fasted group had a significantly greater density in the arm and a significantly lower density in the center area in the no-shelter environment.

### 3.3. Effects of Simulated Predation Attack, Fasting and Shelter on Distribution and Dynamics

**Figure 4:**
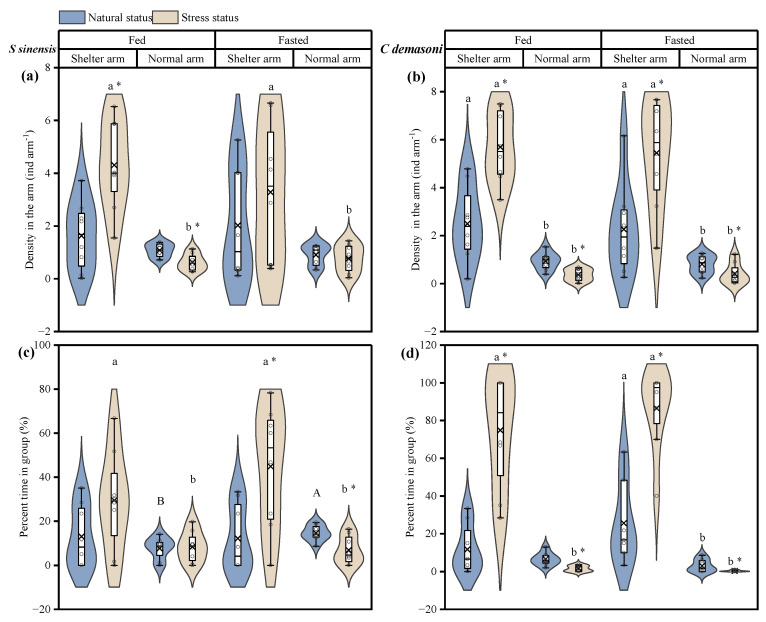
Effects of simulated attack, fasting and arm type on distribution and dynamics of *Spinibarbus sinensis* (**a**,**c**) and *Chindongo demasoni* (**b**,**d**). This figure is a box diagram plus a violin diagram. The black circle (o) indicates the data. The fork (×) indicates the mean of the group. The black horizontal line (―) indicates the median of the group. The widening part of the violin diagram indicates that more data are present in this area. * indicates significant differences before and after the simulated predator attack (*p* < 0.05); a, b indicate significant differences with and without shelter (*p* < 0.05); A, B indicate significant differences between the fed and fasted groups (*p* < 0.05).

In this study, we only compared the density of the distribution and the percentage of time spent in the group in the shelter arm and the normal arm, as little grouping occurred in the food arm after the simulated predation attack in demasone cichlid. The simulated predation attack resulted in increased densities in both species in the shelter and decreased densities in the normal arm (Table 3, Figure 4a–d). However, this phenomenon was present in both the fed and fasted groups for demasone cichlid but only in the fed group for qingbo. The density of both species was generally greater in the shelter arm than in the normal arm, whereas the density of qingbo did not significantly differ between the two arms before the simulated predation attack. There was no significant difference in the percentage of time spent in the group before the simulated predation attack between the fed and fasted groups of qingbo. In qingbo, the density increased significantly in the shelter arm and decreased significantly in the normal arm after the simulated predation attack. As a result, the density of both the qingbo fasted and fed groups in the shelter arm was significantly greater than that in the normal arm after the simulated predation attack. In the demasone cichlid group, the percentage of time in group in the shelter arm increased significantly in both the fed and fasted groups after the simulated predation attack and decreased significantly in the normal arm, and the demasone cichlid group spent a significantly greater percentage of time in the shelter arm than in the normal arm, with the exception of the fed group before the simulated predation attack.

## 4. Discussion

Group behavior in fish is often considered to be influenced by a variety of factors (e.g., environment type, energy metabolism, trophic status and predation pressure and community rank of individuals) [30,31]. We found that qingbo’s group cohesion was unaffected by the simulated predator attack in the two environments (with and without shelter) under different trophic states. The frequency of grouping was not affected by fasting in the absence of shelter. However, both simulated predator attacks and fasting reduced the frequency in the presence of shelter. Meanwhile, the duration was not affected by the simulated predation attack and shelter, and the interaction of shelter and fasting significantly increased the duration. The swimming frequency of the qingbo group was not affected by fasting, shelter or the simulated predation attack. In a previous study on the variation in the group structure of qingbo, it was found that the spatial location of individual members of the group was not affected by the type of environment or the metabolic phenotype [32], which is also reflected in the results of the present study, suggesting that the qingbo group structure may be more influenced by shelter. The individual effects of predation stress and the trophic status may be less pronounced, but their interactions still had an impact on the qingbo group. However, the performance of the demasone cichlid group differed from that of the qingbo group. Without the shelter, the group cohesion of demasone cichlid in both the fasted and fed groups increased significantly after the simulated predation attack. In addition, the duration and swimming frequency of the demasone cichlid group were significantly affected by the simulated predation attack, especially in the fasted group, where the duration and swimming frequency of this group increased significantly regardless of the shelter and remained unchanged only in the fed group. In addition, the percentage of time spent in moving the group was greater after the simulated predation attack in the fed group in the sheltered environment, and the swimming frequency was greater for the fed groups without shelter. This finding suggests that, regardless of the presence or absence of shelter, the fasted demasone cichlid groups became more cohesive after the simulated predation attack and the fasted demasone cichlid groups behaved more cohesively in both the sheltered and non-sheltered environments. Previous studies have proposed that the trophic status influences group behavior in shoals, with group members competing for food resources resulting in increased group swimming activity and greater inter-individual distances [15]. However, the results of our study are different, possibly because fasting leads to a decrease in the glycogen and fat content in tissue types such as the liver and muscle, which cannot provide additional high-energy substances such as phosphocreatine and adenosine triphosphate to support fast-start swimming; this is also consistent with the performance of other fish species [33]. For example, the maximum linear velocity (*V*_max_) was significantly reduced in starved *Channa argus* and *Pseudorasbora parva*, and the sustained acceleration (*U*_cat_) was significantly reduced in starved crucian carp (*Carassius carassius*) [34]. We believe that this reduction in locomotor ability is quite detrimental to predator avoidance, leading to selection among group members to move closer to the center of the group to avoid predation. Overall, although both the qingbo and demasone cichlid groups exhibited good stability, the factors affecting their group structures differed. The qingbo group had better structural stability than the demasone cichlid group, with the qingbo being more motile [35] and less affected by fasting but vulnerable to shelter, whereas the structure of the demasone cichlid group was influenced by a wider range of factors, such as shelter, the trophic status and predation stress.

There was also a difference in the distribution positions of the qingbo and demasone cichlid groups. The distribution position of the qingbo groups was not affected by predation in the no-fasted state but was affected by shelter, and, when shelter was present, the qingbo groups were more likely to occur in the arms than the center of the area. There was no significant difference in the distribution of the qingbo groups between the arm and center areas in the fasted group, but they tended to be more common in the arm after the predator attack. In the demasone cichlid, the fasted group tended to prefer the center area of the maze more than the fed group in the sheltered environment but tended to choose the arm area after the simulated predation attack. Interestingly, when faced with predation in an environment without shelter, the fed group of demasone cichlid chose to move to the center area rather than hide in the arm, and, when shelter was present in the environment, the fed group still chose to hide in the arm after the simulated predation attack. The behavioral responses of qingbo to southern catfish (*Clarias fuscus*) stress were found to be pronounced, with a reduced group activity time and dwell time in danger areas, especially under high predation pressure [36]. In the absence of predators, fasted groups typically exhibit more predator-checking behavior (i.e., approaching predators); however, in the presence of predators, fasted groups check more frequently but for shorter durations, possibly as a result of a trade-off between their energy needs and the predation risk [37], a change that is thought to reduce the likelihood of being detected by predators [38,39,40]. The qingbo groups in this study all tended to stay around the shelter when it was present in the environment, especially for the fed group, and the fed group did not change its group distribution position as a result of the simulated predation attack. This may be because the habitats of qingbo include mostly flowing water environments with strong swimming locomotion, and their ability to respond to a simulated predation attack without starvation, where the threat level is not particularly high, is adequate, so they may not necessarily need to seek refuge in shelter. Furthermore, whereas previous studies have shown that demasone cichlid quickly moved to shelter after encountering a predator stimulus [41], the present study revealed that the fasted group preferred to congregate in the central area of the maze after the simulated predator attack. This is in contrast to the findings of the previous study, and we believe that the reduced locomotor ability of the fasted demasone cichlid group members prevented them from quickly moving to the shelter to escape; instead, they moved to the center area to form a large, tightly knit group to deal with the predator.

In terms of group changes in the specific arms, little group formation occurred in the food arm of the demasone cichlid group after the simulated predation attack. This may indicate that predator avoidance is still a greater priority than feeding, even under starvation conditions. Overall, both the fasted and fed qingbo groups were still more likely to form groups in the shelter to escape predators and to stay in groups for longer periods of time, and the grouping frequency and duration of the fasted groups were diametrically opposed to those of the normal arm in terms of their grouping frequency and duration in the shelter arm as affected by the predator attack. However, both the fasted and fed demasone cichlid groups tended to enter the shelter when attacked by predators, and their durations significantly increased in the shelter arm and significantly decreased in the normal arm. This suggests that in the environment with shelter, demasone cichlid chose to form a group and disperse into the normal arms after being attacked by the predator. It has been suggested that groups of animals typically move more tightly together when faced with predators and more loosely when free foraging [8,9], and our results also support this theory. Moreover, in terms of the variation in group position, we found that the strategy of demasone cichlid groups facing predators in the environment without shelter was to congregate towards the center of the maze. This may indicate a significant difference in motivation between individual members of the group, leading to a change in group structure, which may be the main reason for the reduction in group formation in the normal arm in the present study [42].

In conclusion, there are interspecific differences in the distributions and dynamics of these two fish groups in response to a simulated predation attack under different trophic states. The group structure of both groups was relatively stable, with the qingbo group being more stable, and the effects of fasting on the qingbo group were not significant, while the demasone cichlid group was more susceptible to the effects of fasting, shelter and the simulated predation attack. Our results suggest that even group-stable fish may shift their anti-predation strategies (i.e., entering a shelter to hide shifts to aggregating in situ into a shoal) when starved, and that the worse the swimming ability of the fish, the more affected they are by starvation. Based on the results of this study, we believe that the locomotor ability is likely to play an important role in the anti-predator behavior of fish. In addition, we believe that the cognitive abilities of different fish species may also influence their anti-predator strategies, and we will continue to investigate the mechanisms of anti-predator behavior in fish in the future.

## Figures and Tables

**Table 1:** Effect of simulated predator attack, fasting and shelter on dynamics of fish groups based on generalized linear mixed model analysis.

	*I_c_*		Grouping Frequency (times/min^−1^)		Duration of Group (s)		Percentage of Time in Group (%)	
	*S. sinensis*	*C. demasoni*	*S. sinensis*	*C. demasoni*	*S. sinensis*	*C. demasoni*	*S. sinensis*	*C. demasoni*
Predation, P	*F*_1,28_ = 0.118 *p* = 0.734	*F*_1,56_ = 32.191 *p* < 0.001 *	*F*_1,28_ = 11.024 *p* = 0.003 *	*F*_1,28_ *=* 1.954 *p* = 0.173	*F*_1,28_ = 11.188 *p* = 0.003 *	*F*_1,28_ = 22.737 *p* < 0.001 *	*F*_1,28_ = 0.624 *p* = 0.436	*F*_1,28_ = 36.843 *p* < 0.001 *
Fasting, F	*F*_1,28_ = 0.818 *p* = 0.374	*F*_1,56_ = 4.494 *p* = 0.038 *	*F*_1,28_ = 1.127 *p* = 0.297	*F*_1,28_ = 14.386 *p* < 0.001 *	*F*_1,28_ = 0.420 *p* = 0.522	*F*_1,28_ = 2.660 *p* = 0.114	*F*_1,28_ = 0.511 *p* = 0.480	*F*_1,28_ = 0.144 *p* = 0.707
Shelter, S	*F*_1,28_ = 0.010 *p* = 0.921	*F*_1,56_ = 2.478 *p* = 0.121	*F*_1,28_ = 5.024 *p* = 0.033 *	*F*_1,28_ = 0.030 *p* = 0.864	*F*_1,28_ = 0.080 *p* = 0.779	*F*_1,28_ = 7.545 *p* = 0.010 *	*F*_1,28_ = 0.624 *p* = 0.436	*F*_1,28_ = 14.208 *p* < 0.001 *
P × F	*F*_1,28_ = 0.320 *p* = 0.576	*F*_1,56_ = 0.004 *p* = 0.951	*F*_1,28_ = 0.110 *p* = 0.742	*F*_1,28_ = 3.231 *p* = 0.083	*F*_1,28_ = 0.544 *p* = 0.467	*F*_1,28_ = 1.927 *p* = 0.176	*F*_1,28_ = 3.109 *p* = 0.089	*F*_1,28_ = 7.808 *p* = 0.009
P × S	*F*_1,28_ = 0.010 *p* = 0.921	*F*_1,56_ = 0.233 *p* = 0.631	*F*_1,28_ = 0.441 *p* = 0.512	*F*_1,28_ = 2.553 *p* = 0.121	*F*_1,28_ = 0.053 *p* = 0.819	*F*_1,28_ = 3.642 *p* = 0.067	*F*_1,28_ = 1.032 *p* = 0.318	*F*_1,28_ = 3.749 *p* = 0.063
F × S	*F*_1,28_ = 1.274 *p* = 0.269	*F*_1,56_ = 0.041 *p* = 0.840	*F*_1,28_ = 2.352 *p* = 0.136	*F*_1,28_ = 0.268 *p* = 0.609	*F*_1,28_ = 2.954 *p* = 0.097	*F*_1,28_ = 0.591 *p* = 0.448	*F*_1,28_ = 3.335 *p* = 0.078	*F*_1,28_ = 0.014 *p* = 0.905
P × F × S	*F*_1,28_ = 5.688 *p* = 0.024 *	*F*_1,56_ = 1.026 *p* = 0.351	*F*_1,28_ = 0.992 *p* = 0.328	*F*_1,28_ = 0.000 *p* = 1.000	*F*_1,28_ = 2.232 *p* = 0.146	*F*_1,27_ = 1.166 *p* = 0.289	*F*_1,28_ = 0.000 *p* = 0.992	*F*_1,28_ = 1.482 *p* = 0.234

* indicates a significant effect (*p* < 0.05).

**Table 2:** Effects of simulated predator attack, fasting and shelter availability on distributions of two experimental fish groups based on generalized linear mixed model analysis.

	Density in the Arm (ind arm^−1^)		Density in the Center Area (ind center^−1^)	
	*S. sinensis*	*C. demasoni*	*S. sinensis*	*C. demasoni*
Predation, P	*F*_1,28_ = 15.088 *p* = 0.001 *	*F*_1,28_ = 0.029 *p* = 0.865	*F*_1,28_ = 15.151 *p* = 0.001 *	*F*_1,28_ = 0.037 *p* = 0.849
Fasting, F	*F*_1,28_ = 1.540 *p* = 0.225	*F*_1,28_ = 0.420 *p* = 0.552	*F*_1,28_ = 1.374 *p* = 0.251	*F*_1,28_ = 0.413 *p* = 0.526
Shelter, S	*F*_1,28_ = 7.076 *p* = 0.013 *	*F*_1,28_ = 11.000 *p* = 0.003 *	*F*_1,28_ = 6.781 *p* = 0.015 *	*F*_1,28_ = 10.874 *p* = 0.003 *
P × F	*F*_1,28_ = 0.010 *p* = 0.921	*F*_1,28_ = 11.633 *p* = 0.002 *	*F*_1,28_ = 0.005 *p* = 0.945	*F*_1,28_ = 11.627 *p* = 0.002 *
P × S	*F*_1,28_ = 0.125 *p* = 0.921	*F*_1,28_ = 14.595 *p* = 0.001 *	*F*_1,28_ = 0.071 *p* = 0.792	*F*_1,28_ = 14.514 *p* = 0.001 *
F × S	*F*_1,28_ = 1.672 *p* = 0.207	*F*_1,28_ = 6.669 *p* = 0.015 *	*F*_1,28_ = 1.448 *p* = 0.239	*F*_1,28_ = 6.581 *p* = 0.016 *
P × F × S	*F*_1,28_ = 0.285 *p* = 0.597	*F*_1,28_ = 1.781 *p* = 0.193	*F*_1,28_ = 0.168 *p* = 0.685	*F*_1,28_ = 1.782 *p* = 0.193

* indicates a significant effect (*p* < 0.05).

**Table 3:** Effect of simulated predator attack, fasting and arm type on density and dynamics of fish shoals based on generalized linear mixed model analysis.

	Density in Arm (ind arm^−1^)		Percentage of Time in Group (%)	
	*S. sinensis*	*C. demasoni*	*S. sinensis*	*C. demasoni*
Predation, P	*F*_1,28_ = 6.257 *p* = 0.018 *	*F*_1,28_ = 28.172 *p* < 0.001 *	*F*_1,28_ = 10.018 *p* = 0.004 *	*F*_1,27.55_ = 87.856 *p* < 0.001 *
Fasting, F	*F*_1,28_ = 0.185 *p* = 0.670	*F*_1,28_ = 0.129 *p* = 0.490	*F*_1,28_ = 1.418 *p* = 0.244	*F*_1,27.98_ = 1.281 *p* = 0.267
Arm type, A	*F*_1,28_ = 26.508 *p* < 0.001 *	*F*_1,28_ = 73.862 *p* < 0.001 *	*F*_1,28_ = 13.698 *p* = 0.001 *	*F*_1,27.98_ = 98.440 *p* < 0.001 *
P × F	*F*_1,28_ = 0.677 *p* = 0.418	*F*_1,28_ = 0.025 *p* = 0.875	*F*_1,28_ = 0.346 *p* = 0.561	*F*_1,27.55_ = 0.002 *p* = 0.967
P × A	*F*_1,28_ = 11.502 *p* = 0.002 *	*F*_1,28_ = 51.944 *p* < 0.001 *	*F*_1,28_ = 17.805 *p* < 0.001 *	*F*_1,27.55_ = 113.310 *p* < 0.001 *
F × A	*F*_1,28_ = 166 *p* = 0.687	*F*_1,28_ = 0.063 *p* = 0.803	*F*_1,28_ = 0.306 *p* = 0.584	*F*_1,27.98_ = 2.697 *p* = 0.112
P × F × A	*F*_1,28_ = 1.683 *p* = 0.205	*F*_1,28_ = 0.035 *p* = 0.852	*F*_1,28_ = 3.431 *p* = 0.075	*F*_1,27.55_ = 0.189 *p* = 0.667

* indicates a significant effect (*p* < 0.05).

## Data Availability

Data from this research can be obtained by emailing the corresponding author.

## References

[1] Creel S., Christianson D. (2008). Relationships between direct predation and risk effects. Trends Ecol. Evol..

[2] Thomson J.S., Watts P.C., Pottinger T.G., Sneddon L.U. (2012). Plasticity of boldness in rainbow trout, Oncorhynchus mykiss: Do hunger and predation influence risk-taking behavior?. Horm. Behav..

[3] Álvarez D., Nicieza A.G. (2003). Predator avoidance behaviour in wild and hatchery-reared brown trout: The role of experience and domestication. J. Fish Biol..

[4] Zhang H.Z., Zhu B.S., Yu L.Y., Liu D.P., Wang F., Lu Y.L. (2021). Selection of shelter shape by swimming crab (*Portunus trituberculatus*). Aquac. Rep..

[5] Zhang H.Z., Zhu B.S., Yu L.Y., Wang F. (2022). Shelter Color Selection of Juvenile Swimming Crabs (*Portunus trituberculatus*). Fishes.

[6] Blake C.A., Andersson M.L., Hulthén K., Nilsson P.A., Brönmark C. (2018). Conspecific boldness and predator species determine predation-risk consequences of prey personality. Behav. Ecol. Sociobiol..

[7] Miller N., Garnier S., Hartnett A.T., Couzin I.D. (2013). Both information and social cohesion determine collective decisions in animal groups. Proc. Natl. Acad. Sci. USA.

[8] Herbert-Read J.E. (2016). Understanding how animal groups achieve coordinated movement. J. Exp. Biol..

[9] Jolles J.W., Boogert N.J., Sridhar V.H., Couzin I.D., Manica A. (2017). Consistent individual differences drive collective behavior and group functioning of schooling fish. Curr. Biol..

[10] Gimeno E., Quera V., Beltran F.S., Dolado R. (2016). Differences in shoaling behavior in two species of freshwater fish (*Danio rerio* and *Hyphessobrycon herbertaxelrodi*). J. Comp. Psychol..

[11] Suriyampola P.S., Shelton D.S., Shukla R., Roy T., Bhat A., Martins E.P. (2016). Zebrafish social behavior in the wild. Zebrafish.

[12] Godin J.G.J., Crossman S.L. (1994). Hunger-dependent predator inspection and foraging behaviours in the threespine stickleback (*Gasterosteus aculeatus*) under predation risk. Behav. Ecol. Sociobiol..

[13] McCue M.D. (2010). Starvation physiology: Reviewing the different strategies animals use to survive a common challenge. Comp. Biochem. Physiol. A.

[14] Liu D.P., Su X.P., Wang F., Zhong D.S., Sun Y.F. (2019). Starvation intensifies the impacts of interspecific interactions on foraging behavior of swimming crab (*Portunus trituberculatus*). Aquaculture.

[15] Hanson K.A., Mauland B.A., Shastri A., Wisenden B.D. (2024). Yellowtail damselfish Chrysiptera parasema can associate predation risk with the acoustic call of a heterospecific damselfish following pairing with conspecific alarm cues. J. Fish Biol..

[16] Mehner T., Wieser W. (1994). Energetics and metabolic correlates of starvation in juvenile perch (*Perca fluviatilis*). J. Fish Biol..

[17] Viana M.T., D’Abramo L.R., Gonzalez M.A., García-Suárez J.V., Shimada A., Vásquez-Peláez C. (2007). Energy and nutrient utilization of juvenile green abalone (*Haliotis fulgens)* during starvation. Aquaculture.

[18] Gao L.J., Chen L.Q., Song B. (2004). Effect of starvation and compensatory growth on feeding growth and body biochemical composition in *Acipenser schrenckii* juveniles. J. Fish. China.

[19] Redpath T.D., Cooke S.J., Suski C.D., Arlinghaus R., Couture P., Wahl D.H., Philipp D.P. (2010). The metabolic and biochemical basis of vulnerability to recreational angling after three generations of angling-induced selec tion in a teleost fish. Can. J. Fish. Aquat. Sci..

[20] Ariyomo T.O., Watt P.J. (2015). Effect of hunger level and time of day on boldness and aggression in the zebra fish *Danio rerio*. J. Fish Biol..

[21] Dar S.A., Srivastava P.P., Varghese T., Gupta S., Gireesh-Babu P., Krishna G. (2018). Effects of starvation and refeeding on expression of ghrelin and leptin gene with variations in metabolic parameters in *Labeo rohita* fingerlings. Aquaculture.

[22] Navarro I., Gutierrez J. (1995). Fasting and starvation. Biochem. Mol. Biol. Fishes.

[23] MacNutt M.J., Hinch S.G., Farrell A.P., Topp S. (2004). The effect of temperature and acclimation period on repeat swimming performance in cutthroat trout. J. Fish Biol..

[24] Smart I.E., Cuthill I.C., Scott-Samuel N.E. (2020). In the corner of the eye: Camouflaging motion in the peripheral visual field. Proc. R. Soc. B.

[25] Ding R.H. (1994). Sichuan Ichthyography.

[26] Li S., Konings A.F., Stauffer J.R. (2016). A revision of the *Pseudotropheus elongatus* species group (Teleostei: Cichlidae) with description of a new genus and seven new species. Zootaxa.

[27] Barluenga M., Stolting K.N., Salzburger W., Muschick M., Meyer A. (2006). Sympatric speciation in Nicaraguan crater lake cichlid fish. Nature.

[28] Xia J.G., Liu X., Huang Y. (2019). The link between chemical alarm cue-induced behavioral responses and personality in Rhodeus ocellatus. Acta Ecol. Sin..

[29] Delcourt J., Miller N.Y., Couzin I.D., Garnier S. (2018). Methods for the effective study of collective behavior in a radial arm maze. Behav. Res. Methods.

[30] Krause J. (1994). Differential fitness returns in relation to spatial position in groups. Biol. Rev. Camb. Philos. Soc..

[31] Krause S., Wilson A.D.M., Ramnarine I.W., Herber-Read J.E., Clément R.J.G., Krause J. (2017). Guppies: Occupy consistent positions in social networks: Mechanisms andconsequences. Behav. Ecol..

[32] Yang Y., Ling H., Fu S.J., Zeng L.Q. (2021). Effects of ecological context and metabolic phenotype on collective behaviour of qingbo *Spinibarbus sinensis*. Acta Ecol. Sin..

[33] Martínez M., Guderley H., Dutil J.D., Winger P.D., He P., Walsh S.J. (2003). Condition, prolonged swimming performance and muscle metabolic capacities of cod *Gadus morhua*. J. Exp. Biol..

[34] Cai L., Fang M., Johnson D., Lin S.M., Tu Z.Y., Liu G.Y., Huang Y.P. (2014). Interrelationships between feeding, food deprivation and swimming performance in juvenile grass carp. Aquat. Biol..

[35] Qiu H.X., Liu W.M., Yan Y.L., Long J., Xie X.J. (2021). Effects of waterborne cadmium exposure on *Spinibarbus sinensis* hepatopancreas and kidney: Mitochondrial cadmium accumulation and respiratory metabolism. Comp. Biochem. Physiol. Part C Toxicol. Pharmacol..

[36] Fu C., Fu S.J., Cao Z.D. (2015). Habitat-specific anti-predator behavior variation among pale chub (*Zacco platypus*) along a river. Mar. Freshw. Behav. Physiol..

[37] Tang Z.H., Wu Q., Fu S.J. (2018). Inspection behaviour and interindividual cooperation in juvenile qingbo: The effects of prior predator exposure and food deprivation. J. Ethol..

[38] Zhao M.G., Feng G.P., Wang H.H., Shen C.C., Fu Y.L., Zhang Y.P., Zhang H.X., Yao Y., Chen J.H., Xu W.K. (2024). The Influence of Shelter Type and Coverage on Crayfish (*Procambarus clarkii*) Predation by Catfish (*Silurus asotus*): A Controlled Environment Study. Animals.

[39] Pohlmann K., Grasso F.W., Breithaupt T. (2001). Tracking wakes: The nocturnal predatory strategy of piscivorous catfish. Proc. Natl. Acad. Sci. USA.

[40] Millidine K.J., Armstrong J.D., Metcalfe N.B. (2006). Presence of shelter reduces maintenance metabolism of juvenile salmon. Funct. Ecol..

[41] Fan J. (2022). The Relationships between Personality and Cognitive Ability and Evasion Ability of Freshwater Fish Species.

[42] Bartolini T., Butail S., Porfiri M. (2015). Temperature influences sociality and activity of freshwater fish. Environ. Fishes.

